# Resolving plasmid structures in *Enterobacteriaceae* using the MinION nanopore sequencer: assessment of MinION and MinION/Illumina hybrid data assembly approaches

**DOI:** 10.1099/mgen.0.000118

**Published:** 2017-06-09

**Authors:** Sophie George, Louise Pankhurst, Alasdair Hubbard, Antonia Votintseva, Nicole Stoesser, Anna E. Sheppard, Amy Mathers, Rachel Norris, Indre Navickaite, Chloe Eaton, Zamin Iqbal, Derrick W. Crook, Hang T. T. Phan

**Affiliations:** ^1^​ Nuffield Department of Clinical Medicine, University of Oxford, Oxford, UK; ^2^​ Division of Infectious Diseases and International Health, Department of Medicine, University of Virginia Health System, Charlottesville, Virginia, USA; ^3^​ The Wellcome Trust Centre for Human Genetics, University of Oxford, Oxford, UK; ^4^​ Addenbrookes Hospital, Cambridge, UK

**Keywords:** plasmid reconstruction, MinION nanopore sequencing, Gram-negative *Enterobactericacae*, plasmid assembly

## Abstract

This study aimed to assess the feasibility of using the Oxford Nanopore Technologies (ONT) MinION long-read sequencer in reconstructing fully closed plasmid sequences from eight *Enterobacteriaceae* isolates of six different species with plasmid populations of varying complexity. Species represented were *Escherichia coli*, *Klebsiella pneumoniae*, *Citrobacter freundii*, *Enterobacter cloacae, Serratia marcescens* and *Klebsiella oxytoca*, with plasmid populations ranging from 1–11 plasmids with sizes of 2–330 kb. Isolates were sequenced using Illumina (short-read) and ONT’s MinION (long-read) platforms, and compared with fully resolved PacBio (long-read) sequence assemblies for the same isolates. We compared the performance of different assembly approaches including SPAdes, plasmidSPAdes, hybridSPAdes, Canu, Canu+Pilon (canuPilon) and npScarf in recovering the plasmid structures of these isolates by comparing with the gold-standard PacBio reference sequences. Overall, canuPilon provided consistently good quality assemblies both in terms of assembly statistics (N50, number of contigs) and assembly accuracy [presence of single nucleotide polymorphisms (SNPs)/indels with respect to the reference sequence]. For plasmid reconstruction, Canu recovered 70 % of the plasmids in complete contigs, and combining three assembly approaches (Canu or canuPilon, hybridSPAdes and plasmidSPAdes) resulted in a total 78 % recovery rate for all the plasmids. The analysis demonstrated the potential of using MinION sequencing technology to resolve important plasmid structures in *Enterobacteriaceae* species independent of and in conjunction with Illumina sequencing data. A consensus assembly derived from several assembly approaches could present significant benefit in accurately resolving the greatest number of plasmid structures.

## Abbreviations

AR, antimicrobial resistance; ONT, Oxford Nanopore Technologies; SNP, single nucleotide polymorphism; WGS, whole-genome sequencing.

## Data Summary

1. Illumina sequencing data and MinION sequencing data have been deposited in NCBI: project accession number PRJNA353060.

2. PacBio assemblies are available from NCBI: accession numbers CAV1411 CP011579–CP011581 (https://www.ncbi.nlm.nih.gov/nuccore/CP011579; https://www.ncbi.nlm.nih.gov/nuccore/CP011580; https://www.ncbi.nlm.nih.gov/nuccore/CP011581).

CAV1374 CP011625–CP011636 (https://www.ncbi.nlm.nih.gov/nuccore/CP011625; https://www.ncbi.nlm.nih.gov/nuccore/CP011626; https://www.ncbi.nlm.nih.gov/nuccore/CP011627; https://www.ncbi.nlm.nih.gov/nuccore/CP011628; https://www.ncbi.nlm.nih.gov/nuccore/CP011629; https://www.ncbi.nlm.nih.gov/nuccore/CP011630; https://www.ncbi.nlm.nih.gov/nuccore/CP011631; https://www.ncbi.nlm.nih.gov/nuccore/CP011632; https://www.ncbi.nlm.nih.gov/nuccore/CP011633; https://www.ncbi.nlm.nih.gov/nuccore/CP011634; https://www.ncbi.nlm.nih.gov/nuccore/CP011635; https://www.ncbi.nlm.nih.gov/nuccore/CP011636).

CAV1492 CP011637–CP011642 (https://www.ncbi.nlm.nih.gov/nuccore/CP011637; https://www.ncbi.nlm.nih.gov/nuccore/CP011638; https://www.ncbi.nlm.nih.gov/nuccore/CP011639; https://www.ncbi.nlm.nih.gov/nuccore/CP011640; https://www.ncbi.nlm.nih.gov/nuccore/CP011641; https://www.ncbi.nlm.nih.gov/nuccore/CP011642).

CAV1596 CP011643–CP011647 (https://www.ncbi.nlm.nih.gov/nuccore/CP011643; https://www.ncbi.nlm.nih.gov/nuccore/CP011644; https://www.ncbi.nlm.nih.gov/nuccore/CP011645; https://www.ncbi.nlm.nih.gov/nuccore/CP011646; https://www.ncbi.nlm.nih.gov/nuccore/CP011647).

CAV1741 CP011651–CP011657 (https://www.ncbi.nlm.nih.gov/nuccore/CP011651; https://www.ncbi.nlm.nih.gov/nuccore/CP011652; https://www.ncbi.nlm.nih.gov/nuccore/CP011653; https://www.ncbi.nlm.nih.gov/nuccore/CP011654; https://www.ncbi.nlm.nih.gov/nuccore/CP011655; https://www.ncbi.nlm.nih.gov/nuccore/CP011656; https://www.ncbi.nlm.nih.gov/nuccore/CP011657).

CAV1015 CP017928–CP017933 (https://www.ncbi.nlm.nih.gov/nuccore/CP011928; https://www.ncbi.nlm.nih.gov/nuccore/CP011929; https://www.ncbi.nlm.nih.gov/nuccore/CP011930; https://www.ncbi.nlm.nih.gov/nuccore/CP011931; https://www.ncbi.nlm.nih.gov/nuccore/CP011932; https://www.ncbi.nlm.nih.gov/nuccore/CP011933).

CAV1016 CP017934–CP017937 (https://www.ncbi.nlm.nih.gov/nuccore/CP011934; https://www.ncbi.nlm.nih.gov/nuccore/CP011935; https://www.ncbi.nlm.nih.gov/nuccore/CP011936; https://www.ncbi.nlm.nih.gov/nuccore/CP011937).

P46212 CP013657–CP013658 (https://www.ncbi.nlm.nih.gov/nuccore/CP013657; https://www.ncbi.nlm.nih.gov/nuccore/CP013658).

## Impact Statement

The long-read MinION sequencer by Oxford Nanopore Technologies (ONT) promises a quick, compact and potentially cost-effective solution to bacterial sequencing. This will be beneficial not only in clinical and epidemiological contexts (e.g. in making medical decisions on antibiotic treatments and in infection control), but also in bacterial genome research (e.g. in understanding bacterial evolution). We assessed the ability of different sequence assembly approaches in assembling and fully resolving plasmid structures from data generated by the MinION and Illumina sequencers for eight *Enterobacteriaceae* isolates from six different species. We selected these isolates to maximize the plasmid population and structural diversity within samples. We compared these assemblies with gold-standard reference sequences generated using PacBio and found that Canu/canuPilon resolved 70 % of the plasmids, outperforming the other hybrid approaches, hybridSPAdes and npScarf, and non-hybrid approaches using Illumina data alone (SPAdes, plasmidSPAdes). Failure to fully resolve plasmid structures using Canu can potentially be attributed to the presence of long repeat structures extending beyond the sequencing read length limits and experimental variability resulting from the use of multiple DNA extracts. Our findings also highlight the need for the development of a meta-assembler to aggregate different methods’ assemblies. Our study is relevant to researchers working in epidemiology, bacterial evolution and clinical practice, especially those working on antimicrobial resistance mechanisms transmitted via mobile genetic elements in general and plasmids in particular.

## Introduction

Plasmids are extra-chromosomal genetic elements that are transmission vectors of many acquired antimicrobial resistance (AR) genes in *Enterobacteriaceae* [1]. The tracking of AR genes and plasmids in clinical outbreaks helps identify transmission routes, potentially informing interventions for future outbreak prevention [2]. Plasmid tracking can be facilitated by the categorization of plasmids using plasmid classification schemes such as incompatibility (Inc), relaxase (MOB) and mating pair formation system (MPF) typing [3]. However, these schemes lack resolution and fail to classify all known plasmids, limiting their application to plasmid transmission epidemiology. Further, linkage between AR genes and plasmid types cannot always be established due to the high frequency of DNA exchange between mobile genetic elements within plasmids [4].

Whole-genome sequencing (WGS) of bacteria and plasmids offers high resolution for tracking clonal outbreaks. However, short-read sequencing is not always suitable for uncovering genetic relationships between isolates in plasmid-mediated AR gene outbreaks [4]. Plasmids often contain many smaller, mobile repeat structures including insertion sequences and transposable elements that enable AR genes to mobilize under evolutionary pressure. These repeat structures often extend beyond the current typical insert size of paired-end short-read sequencing (~300–500 bp), and therefore inhibit complete plasmid assembly using short-reads, meaning the AR genes cannot be readily contextualized. Isolating and sequencing individual plasmids of interest by electroporation can improve assemblies by removing repeats also present on other replicons, but this approach remains limited if multiple copies of the same repeat unit are present on the electroporated plasmid. Furthermore, electroporation is time-consuming and not practical for large-scale projects.

Long-read sequencing such as PacBio’s SMRT technology can address some of these shortcomings. Several studies have used PacBio sequencing for plasmid tracking and characterization [2, 5, 6]. However, barriers to the widespread use of PacBio sequencing lie in the prohibitive cost of sequencing large numbers of isolates.

Oxford Nanopore Technologies’ (ONT) MinION, a compact and rapid long-read sequencer, could offer an effective alternative. Its small size and high sequencing speed mean that it could potentially be used within clinical settings. However, the relatively high error rate of MinION sequencing data [7] could hinder high-resolution tracking of AR genes and their mobile vectors (e.g. plasmids, transposons). Although increased MinION sequencing coverage can improve accuracy, it is not always technically achievable and/or is not equivalent to the accuracy achieved with short-read (Illumina) methods. One solution to this is to combine MinION data with highly accurate Illumina data using hybrid assembly approaches.

The use of MinION reads for bacterial genome assembly has been explored in several studies [8–14]; however, these either did not focus on accurate plasmid sequence reconstruction or have only included 1–2 bacterial isolates. In this study, we assessed the efficiency of plasmid reconstruction for six different *Enterobacteriaceae* species using Illumina short-read and MinION long-read data, compared with ‘gold-standard’ reference genome assemblies created using PacBio long-read data. We explored the strengths and weaknesses of different assemblers using both overall and plasmid-specific assembly statistics. Approaches studied included the widely used SPAdes [15] assemblers using either Illumina data alone (SPAdes, plasmidSPAdes) or in combination with MinION data (hybrid SPAdes); the contig scaffolder npScarf [16], using MinION data to scaffold SPAdes assemblies; and the long-read assembler Canu for MinION data [17], with the option of assembly polishing with short-read data using pilon [18]. Running times for the assemblers were comparable to those previously reported and are not discussed in this study [10, 19].

## Methods

### Isolate selection

Isolates with associated, pre-existing Illumina and PacBio sequencing data (see Data Summary) were selected from local collections [5, 20]. In order to maximize the diversity of plasmid profiles, we chose eight isolates representing six *Enterobacteriaceae* species, namely *Klebsiella pneumoniae* (*Kpne*, *n*=2), *Escherichia coli* (*Ecol*, *n*=1), *Klebsiella oxytoca* (*Koxy*, *n=*2), *Citrobacter freundii* (*Cfre*, *n=*1), *Enterobacter cloacae* (*Eclo*, *n*=1) and *Serratia marcescens* (*Smar, n=*1). The isolates contained 1–11 distinct plasmids (2–330 kb in size), some of which had large structural repeats (Table S1, available in the online Supplementary Material). Seven isolates harboured the *bla_KPC_* gene nested within the 10 kb Tn*4401* transposon as previously described [21]. One contained two unique Tn*4401-*carrying plasmids, and one contained two copies of Tn*4401* on a single plasmid.

### DNA extraction and sequencing

Isolates were cultured from frozen stocks (−80 °C) on MacConkey agar overnight at 37 °C, sweeps taken from across the culture plate and genomic DNA isolated using the Qiagen Genomic-tip 100/G kit (Qiagen) following the manufacturer’s recommendations. DNA was quantified using the Qubit 2.0 Fluorometer (Life Technologies), and fragment length was assessed using the TapeStation 2200 (Agilent).

### DNA fragmentation

Fragmentation was performed using Covaris G-tubes (Covaris), with 4 µg DNA in a 46 µl volume centrifuged at 4200–5000 r.p.m. (Eppendorf 5424 centrifuge) for 90 s to achieve fragment sizes of ~20 kb.

### MinION sequencing

Library preparations were performed using a SQK-LSK208 Ligation Sequencing 2D kit with and without Native Barcoding (ONT) according to the manufacturer’s protocol (Supplementary Methods). Libraries were loaded onto flow cell versions FLO-MIN106 R9.4 SpotON and sequenced for 48 h. Flow cells were restarted between 12 and 24 h into the sequencing run. Base calling was performed in real-time via ONT’s Metrichor service (desktop agent v2.43.1, 2D-basecalling with/without barcoding workflow v1.125).

### Genomics analysis

We applied six assembly approaches to short-read Illumina and/or long-read MinION sequencing data: (1) SPAdes (v3.10) [15] using only Illumina data; (2) Canu [17] (v1.4) using MinION data; (3) canuPilon (Canu assembly polished by Illumina data using pilon [18] v1.18); (4) hybridSPAdes (v3.10) using Illumina and MinION data; (5) the npScarf pipeline [16] (downloaded June 2016) to scaffold the SPAdes assemblies in (1) using MinION reads; and (6) plasmidSPAdes [22] (v3.10) for plasmid assembly using only Illumina data (Supplementary Methods). We converted MinION 2D fast5 format reads that had passed Metrichor quality control into fasta and fastq format using poretools [23]. We compared the assemblies with the PacBio-generated references using dnadiff [24], a wrapper of nucmer which aligns two similar genomes and renders reports on the alignment statistics, single nucleotide polymorphisms (SNPs), breakpoints, etc. between them.

We assessed each method’s performance using standard assembly quality metrics (assembly size, total number of contigs, N50, maximum contig length, mean contig length) and by its ability to fully resolve plasmid structures. A plasmid was defined as fully resolved when its contig could: (1) be circularized (i.e. contained overlapping ends >100 bp, 100 % identity for short-read sequence based approaches and >1000 bp, >99 % identity for Canu-based approaches); and (2) was syntenically consistent with the corresponding PacBio plasmid structure (Supplementary Methods).

## Results

### MinION sequencing read statistics

MinION sequencing yielded a lower mean coverage than Illumina sequencing (~50× versus ~110×, respectively). MinION sequencing yields ranged from 291.3 to 809.1 Mb. After de-multiplexing, per-sample yield ranged from 89.1 to 809.1 Mb, translating to estimated coverage depths of 15.3–122.9× (Table 1). Mean read lengths across the eight samples ranged from 8.8–12.5 kb, with a consistently high number of reads >10 kb (~7–43 kb, Table 1 and Fig. S1).

**Table 1.** Summary statistics of 8 MinION long-read sequencing runs based on 2D reads that passed quality control

Sample	CAV1015	CAV1016	CAV1374	CAV1411	CAV1492	CAV1596	CAV1741	P46212
Sequencing run	1	2	3	3	4	4	5	5
Estimated coverage	122.92	77.58	36.58	36.49	15.29	57.31	25.37	29.43
Total bases (Mb)	809.13	434.22	264.38	182.65	89.12	322.09	136.77	154.49
No. 2D reads	69 806	42 889	29 841	18 013	7 044	29 251	10 966	13 055
Mean read length (bp)	11 591	10 124	8 860	10 140	12 653	11 011	12 472	11 834
Max. read length (bp)	43 246	67 060	49 368	57 507	57 649	69 030	36 407	45 085
N50 (bp)	14 288	13 021	11 939	12 209	17 593	16 347	15 481	15 065
*N*>20 kb	5 591	2 544	905	515	1 326	4 121	1 212	1 418
*N*>10k	43 013	20 392	12 621	9 501	4 184	14 797	7 330	7 899
*N*>5k	58 660	33 592	20 540	14 740	5 317	19 576	9 098	10 403
*N*<1k	943	476	321	88	91	582	21	25

### Whole-genome assembly assessments

**Fig. 1.**
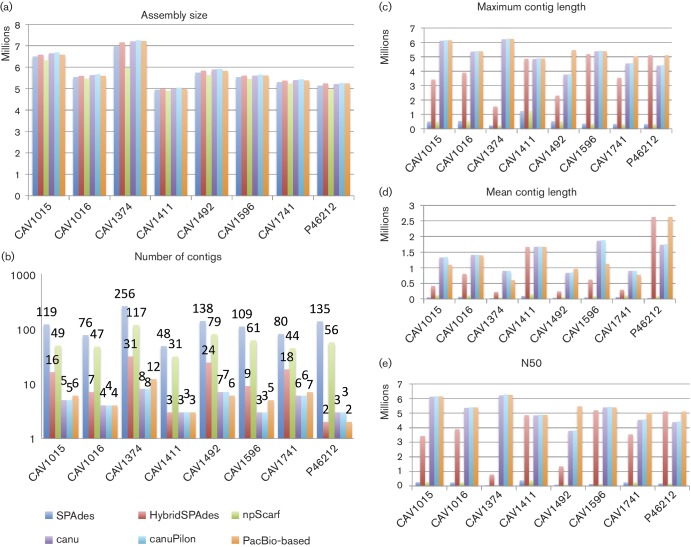
Assembly summary statistics for sequences including assembly size (a), number of contigs (b), maximum contig size (c), mean contig length (d) and N50 (e) – the results of plasmidSPAdes are not included as it is not a complete genome assembly method. SPAdes used only Illumina short-read data, Canu used only MinION long-read data, whereas hybridSPAdes, npScarf and canuPilon used both.

Among WGS assemblers, those utilizing MinION data (Canu, canuPilon, hybridSPAdes and npScarf) showed clear improvements in assembly statistics (longer and fewer contigs; Fig. 1). Chromosomal contigs matching >95 % of the reference chromosomal sequence length were achieved in 7/8 cases, either by hybridSPAdes (CAV1411, P46212) or Canu/canuPilon (CAV1015, CAV1016, CAV1374, CAV1411, CAV1596, CAV1741). Most of these long chromosomal contigs were syntenically consistent with the reference assemblies (Figs S2a, b, S3 and S4) apart from a mis-assembly error for CAV1492’s Canu assembly (Figs S2b and S3). In all cases, MinION data enabled the resolution of gaps in the Illumina-only assemblies resulting from the presence of long repeat structures.

Overall, Canu-based approaches performed consistently well across all samples in terms of assembly metrics such as contig numbers, N50 and contig length, with better assembly statistics on 7/8 occasions. However, on four of these occasions, Canu-based assemblies had fewer contigs than the gold-standard references (CAV1015, CAV1374, CAV1596 and CAV1741; see Plasmid assembly assessment). HybridSPAdes was better than the baseline SPAdes approach and npScarf. HybridSPAdes outperformed Canu-based approaches in one case (P46212) where it produced an assembly with two contigs that were structurally identical to the PacBio-based reference.

**Fig. 2.**
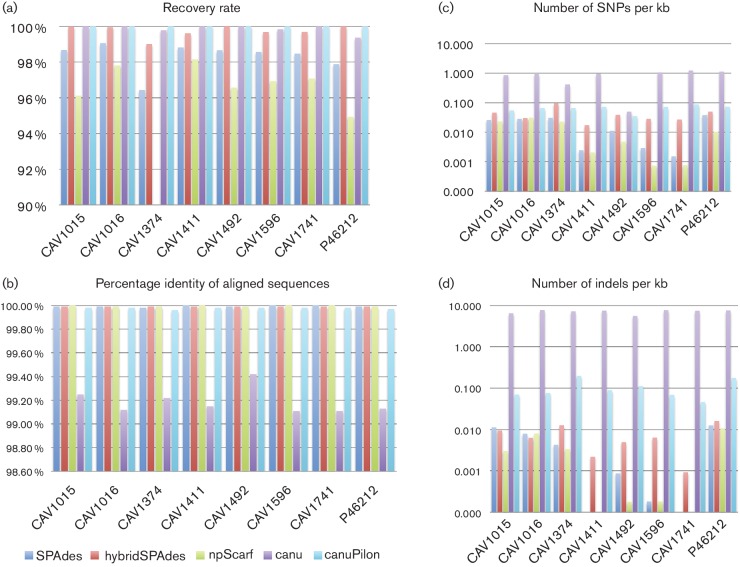
Comparison of assemblies with the PacBio reference genomes – the results of plasmidSPAdes are not included as it is not a complete genome assembly method.

In terms of SNP/indel-level accuracy, SPAdes, hybridSPAdes and npScarf generally performed best, with far fewer SNP differences and indels (0.0007–0.09 SNPs per kb, 0–0.015 indels per kb) compared with Canu using MinION reads only (0.049–1.22 SNPs per kb, 5.62–7.68 indels per kb) (Fig. 2). Polishing assemblies (canuPilon) resulted in lower SNP (0.03–0.08 SNPs per kb) and indel error rates (0.05–0.20 indels per kb). Increased sequencing depth did not clearly correlate with increased assembly accuracy [e.g. CAV1492 (15x), 0.049 SNPs per kb; CAV1015 (122x), 0.86 SNPs per kb]. The Canu assemblies suffered from observable SNP change biases as detected by dnadiff, with A>G and T>C transition changes occurring most often [36.5 % A>G and 33.3 % T>C mutations (mean 8.3 and 7.1 %, respectively, for canuPilon assemblies)].

### Plasmid assembly assessment

Canu/canuPilon outperformed the other assemblers in resolving complete plasmid structures. It resolved 26/37 (70 %) of the plasmids present, better than hybridSPAdes (8/37, 22 %), followed by SPAdes and plasmidSPAdes (5/37, 14 %) (Table 2). npScarf performed worst with only 4/37 (11 %) plasmids recovered.

**Table 2.** Summary of plasmid structure recovery of different assembly approaches using Illumina and/or MinION long-read sequences

	CAV1015	CAV1016	CAV1374	CAV1411	CAV1492	CAV1596	CAV1741	P46212	Total number of plasmids recovered	Recovery rate
Number of plasmids (reference PacBio assembly)	5 (a–e)	3 (a–c)	11 (a–k)	2 (a, b)	5 (a–e)	4 (a–d)	6 (a–f)	1 (a)	37	
SPAdes*	1, c	1, a	0	0	0	1, a	2 b, e	0	5	14 %
plasmidSPAdes*	2 a,c	1, a	0	0	0	0	2	0	5	14 %
Hybrid SPAdes†	1, c	1, a	0	0	1, c	1, a	2 b, e	1	7	19 %
npScarf†	1, c	1, a	0	0	0	0	1, e	1	4	11 %
Canu/canuPilon†	4, b–e	3, a-c	6, c, f–i, k	2, a, b	5, a–e	3 b–d	2, d, e	1	26	70 %
Aggregated results	5	3	6	2	5	4	3	1	29	78 %

*Assembly using Illumina short-read data only.

†Hybrid assembly – Illumina short-read plus MinION long-read data.

Failure of Canu/canuPilon to resolve some plasmids was likely multi-factorial, potentially involving plasmid loss during culturing and DNA extraction processes and/or plasmid sequence complexity. Canu/canuPilon failed to recover 11/37 plasmids, nine of which were small (<35 kb). Re-mapping long-reads to these small plasmids demonstrated reasonable (20–140x) coverage for those in CAV1015, CAV1374 and CAV1596 (Fig. S7). This indicates the failure of the Canu assembler to assemble these reads into contigs, likely due to repeat regions in these plasmids being present elsewhere in the genomes, shown by elevated coverage in these regions on the plasmids and confirmed by self-self blasting of the relevant PacBio reference genomes. Meanwhile, coverage for small plasmids in CAV1741 was <5×, indicating plasmid loss during culture and extraction for this study, or during sample preparation and MinION sequencing itself.

**Fig. 3.**
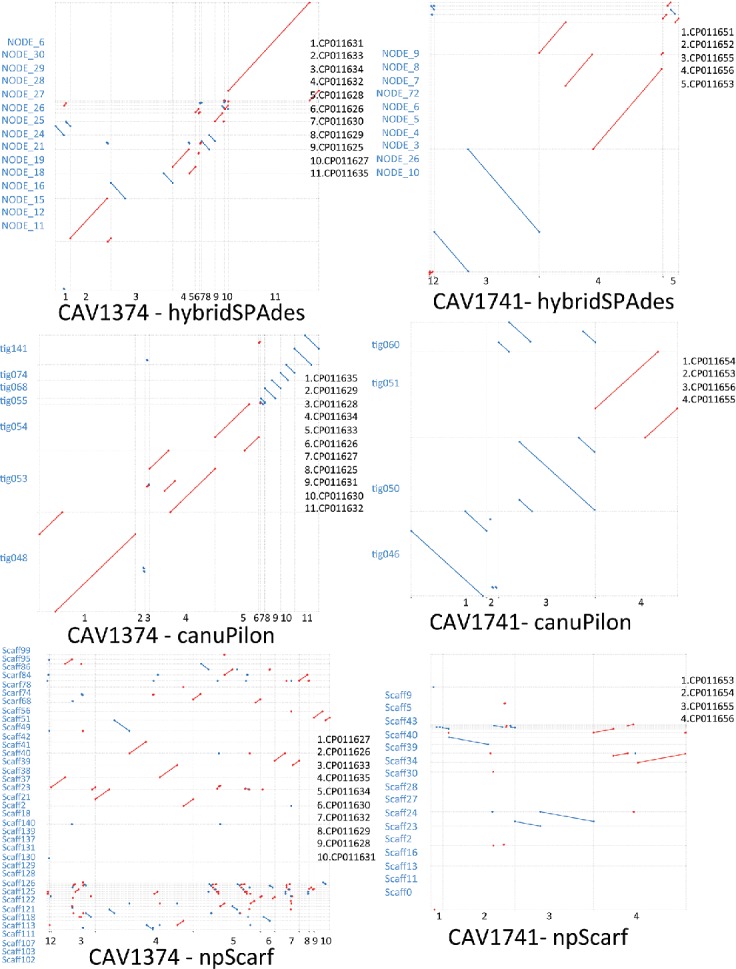
Mummerplots comparing study plasmid assemblies and reference plasmid sequences, for canuPilon, hybridSPAdes and npScarf approaches, and for isolates CAV1374, CAV1741 (x-axis: reference plasmids; y-axis: matched contigs from assemblies; red, sequence match in the forward direction; blue, matching in the reverse complement direction; legends of x-axis on the right of subplots ordering by left to right, legends of y-axis on the left of subplots in blue, ordering up to down). A resolved plasmid is indicated by overlapping ends, shown as overlapping co-ordinates of diagonal lines on the x-axis. The number of reference plasmids in the subplots differs between assembly methods due to the missing of contigs matched with any of these reference plasmids from the assemblies. The mummerplot comparisons of other samples are in Figs S6 and S7.

On the two occasions where Canu failed to resolve large plasmids, one plasmid [CAV1741(e); 330 kb] has two Tn*4401* elements nested within another transposon structure [5], resulting in 15 kb repeats on the same plasmid, and one plasmid [CAV1374(j); 227 kb] contains a 35 kb repeat structure at two locations (52–87 kb, 148–183 kb) (Fig. 3 and S8). The lengths of these repeat structures are well beyond the range of the current MinION sequencing read length obtained by this study (mean ~10 kb), and this explains why these plasmids could not be resolved.

Importantly, the aggregation of Canu, hybridSPAdes and plasmidSPAdes approaches brought the number of completely resolved plasmids to 29/37 (78 %), with one additional plasmid from plasmidSPAdes [CAV1015(a), 11.6 kb] and two from hybridSPAdes [CAV1596(a), 3 kb; CAV1741(b), 3 kb].

## Conclusions

MinION sequencing was beneficial in resolving plasmid structures for the *Enterobacteriaceae* species studied. The technology did not offer consistent sequencing yield in this investigation. However, interestingly, sequencing coverage did not seem to influence successful plasmid assembly or assembly accuracy. Here, 2D MinION sequencing was used owing to the higher accuracy offered (96 %). 1D MinION sequencing is lower accuracy (92 %), but offers the potential for increased multiplexing owing to higher yields, as well as alternative base calling algorithms that may improve accuracy and bias in the future (for example, Albacore and Nanonet). In-house costings (data not shown) for PacBio and MinION sequencing (excluding capital costs and labour) are currently equivalent; and additional multiplexing of samples for MinION sequencing would further reduce cost per sample and improve scalability.

Canu-based approaches were the best individual approach facilitating recovery of complete plasmid structures, although the assemblies were of lower quality and accuracy overall when only long-reads were used. However, the best plasmid recovery rates were obtained by combining three approaches (Canu, hybridSPAdes and plasmidSPAdes) and might be desirable in future analyses; these meta-assembly approaches must be designed with care to avoid aggregating assembly errors that might be inherent within the individual assembly approaches.

The assembly of complex plasmid structures due to long repeats remains difficult. From the MinION read length distributions in this study (Fig. S1), large repeat structures (e.g. the Tn*4401*-habouring 15 kb repeat or the 35 kb repeat mentioned above) pose problems even with increased coverage. Therefore, sample preparation protocols enriching for longer reads [25, 26] or DNA preparation without fragmentation may be useful. Increasing fragment size or sequencing unfragmented DNA also increases the likelihood that very low numbers of high-quality 2D reads are generated [25], potentially decreasing coverage overall. Care also needs to be taken to avoid depletion of small plasmids that may carry important AR genes. Future work should focus on improving DNA fragment size distributions without being detrimental to overall coverage in order to improve plasmid resolution for outbreak investigations, and to consider deployment of this tool in ‘real-time’ outbreak analysis.

## Data bibliography

1. Sheppard AE, Stoesser N, Wilson D, Sebra R, Kasarskis A *et al*. NCBI. CAV1411 CP011579~CP011581 CAV1374 CP011625~CP011636 CAV1492 CP011637~CP011642 CAV1596 CP011643~CP011647 CAV1741 CP011651~CP011657 CAV1015 CP017928~CP017933 CAV1016 CP017934~CP017937 P46212 CP013657~CP013658 (2015).
2. George S, Pankhurst L, Hubbard A, Votintseva A, Stoesser N *et al*. SRA. PRJNA353060 (2016).

## Supplementary Data

5830 Supplementary File 1

## References

[1] Partridge SR (2015). Resistance mechanisms in *Enterobacteriaceae*. Pathology.

[2] Conlan S, Thomas PJ, Deming C, Park M, Lau AF (2014). Single-molecule sequencing to track plasmid diversity of hospital-associated carbapenemase-producing *Enterobacteriaceae*. Sci Transl Med.

[3] Shintani M, Sanchez ZK, Kimbara K (2015). Genomics of microbial plasmids: classification and identification based on replication and transfer systems and host taxonomy. Front Microbiol.

[4] Sheppard AE, Stoesser N, Wilson DJ, Sebra R, Kasarskis A (2016). Nested Russian doll-like genetic mobility drives rapid dissemination of the carbapenem resistance gene *bla*_KPC_. Antimicrob Agents Chemother.

[5] Mathers AJ, Stoesser N, Sheppard AE, Pankhurst L, Giess A (2015). *Klebsiella pneumoniae* carbapenemase (KPC)-producing *K. pneumoniae* at a single institution: insights into endemicity from whole-genome sequencing. Antimicrob Agents Chemother.

[6] Conlan S, Park M, Deming C, Thomas PJ, Young AC (2016). Plasmid dynamics in KPC-positive *Klebsiella pneumoniae* during long-term patient colonization. MBio.

[7] Wang Y, Yang Q, Wang Z (2014). The evolution of nanopore sequencing. Front Genet.

[8] Turton JF, Doumith M, Hopkins KL, Perry C, Meunier D (2016). Clonal expansion of *Escherichia coli* ST38 carrying a chromosomally integrated OXA-48 carbapenemase gene. J Med Microbiol.

[9] Judge K, Harris SR, Reuter S, Parkhill J, Peacock SJ (2015). Early insights into the potential of the Oxford Nanopore MinION for the detection of antimicrobial resistance genes. J Antimicrob Chemother.

[10] Judge K, Hunt M, Reuter S, Tracey A, Quail MA (2016). Comparison of bacterial genome assembly software for MinION data and their applicability to medical microbiology. Microb Genom.

[11] Loman NJ, Quick J, Simpson JT (2015). A complete bacterial genome assembled *de novo* using only nanopore sequencing data. Nat Methods.

[12] Risse J, Thomson M, Patrick S, Blakely G, Koutsovoulos G (2015). A single chromosome assembly of *Bacteroides fragilis* strain BE1 from Illumina and MinION nanopore sequencing data. Gigascience.

[13] Ashton PM, Nair S, Dallman T, Rubino S, Rabsch W (2015). MinION nanopore sequencing identifies the position and structure of a bacterial antibiotic resistance island. Nat Biotechnol.

[14] Deschamps S, Mudge J, Cameron C, Ramaraj T, Anand A (2016). Characterization, correction and *de novo* assembly of an Oxford Nanopore genomic dataset from *Agrobacterium tumefaciens*. Sci Rep.

[15] Bankevich A, Nurk S, Antipov D, Gurevich AA, Dvorkin M (2012). SPAdes: a new genome assembly algorithm and its applications to single-cell sequencing. J Comput Biol.

[16] Cao MD, Nguyen SH, Ganesamoorthy D, Elliott AG, Cooper MA (2017). Scaffolding and completing genome assemblies in real-time with nanopore sequencing. Nat Commun.

[17] Koren S, Walenz BP, Berlin K, Miller JR, Bergman NH (2017). Canu: scalable and accurate long-read assembly via adaptive *k*-mer weighting and repeat separation. Genome Res.

[18] Walker BJ, Abeel T, Shea T, Priest M, Abouelliel A (2014). Pilon: an integrated tool for comprehensive microbial variant detection and genome assembly improvement. PLoS One.

[19] Sović I, Križanović K, Skala K, Šikić M (2016). Evaluation of hybrid and non-hybrid methods for de novo assembly of nanopore reads. Bioinformatics.

[20] Stoesser N, Sheppard AE, Pankhurst L, De Maio N, Moore CE (2016). Evolutionary history of the global emergence of the *Escherichia coli* epidemic clone ST131. MBio.

[21] Cuzon G, Naas T, Nordmann P (2011). Functional characterization of Tn*4401*, a Tn*3*-based transposon involved in *bla*_KPC_ gene mobilization. Antimicrob Agents Chemother.

[22] Antipov D, Hartwick N, Shen M, Raiko M, Lapidus A (2016). plasmidSPAdes: assembling plasmids from whole genome sequencing data. Bioinformatics.

[23] Loman NJ, Quinlan AR (2014). Poretools: a toolkit for analyzing nanopore sequence data. Bioinformatics.

[24] Kurtz S, Phillippy A, Delcher AL, Smoot M, Shumway M (2004). Versatile and open software for comparing large genomes. Genome Biol.

[25] Urban JM, Bliss J, Lawrence CE, Gerbi SA (2015). Sequencing ultra-long DNA molecules with the Oxford Nanopore MinION. bioRxiv.

[26] Eckert SE, Chan JZ-M, Houniet D, Breuer J, the PATHSEEK consortium (2016). Enrichment by hybridisation of long DNA fragments for Nanopore sequencing. Microb Genom.

