# Corrigendum: Resolving plasmid structures in *Enterobacteriaceae* using the MinION nanopore sequencer: assessment of MinION and MinION/Illumina hybrid data assembly approaches

**DOI:** 10.1099/mgen.0.000130

**Published:** 2018-03-29

**Authors:** Sophie George, Louise Pankhurst, Alasdair Hubbard, Antonia Votintseva, Nicole Stoesser, Anna E. Sheppard, Amy Mathers, Rachel Norris, Indre Navickaite, Chloe Eaton, Zamin Iqbal, Derrick W. Crook, Hang T. T. Phan

**Affiliations:** ^1^​ Nuffield Department of Clinical Medicine, University of Oxford, Oxford, UK; ^2^​ Division of Infectious Diseases and International Health, Department of Medicine, University of Virginia Health System, Charlottesville, Virginia, USA; ^3^​ The Wellcome Trust Centre for Human Genetics, University of Oxford, Oxford, UK; ^4^​ Addenbrookes Hospital, Cambridge, UK

There was an error in the Data Summary in the published article. PacBio assembly numbers beginning with CP0119XX should be CP0179XX, as shown below.

CAV1015 CP017928–CP017933 (https://www.ncbi.nlm.nih.gov/nuccore/CP017928; https://www.ncbi.nlm.nih.gov/nuccore/CP017929; https://www.ncbi.nlm.nih.gov/nuccore/CP017930; https://www.ncbi.nlm.nih.gov/nuccore/CP017931; https://www.ncbi.nlm.nih.gov/nuccore/CP017932; https://www.ncbi.nlm.nih.gov/nuccore/CP017933).

CAV1016 CP017934–CP017937 (https://www.ncbi.nlm.nih.gov/nuccore/CP017934; https://www.ncbi.nlm.nih.gov/nuccore/CP017935; https://www.ncbi.nlm.nih.gov/nuccore/CP017936; https://www.ncbi.nlm.nih.gov/nuccore/CP017937).

The author apologizes for any inconvenience.

