# Supplementary material for: Resolving plasmid structures in Enterobacteriaceae using the MinION nanopore sequencer: assessment of MinION and MinION/Illumina hybrid data assembly approaches
Source: Microb Genom. 2017 Jun 9;3(8):e000118. doi: 10.1099/mgen.0.000118 (PMC5610714; doi:10.1099/mgen.0.000118)
Supplement: Supplementary File 1 [file mgen-3-118-s001.pdf]

Resolving plasmid structures in Enterobacteriaceae using the MinION nanopore sequencer: assessment of MinION and MinION/Illumina hybrid data assembly approaches

Supplementary

Table S1. Summary of samples selected in the study including species, reference assembly information and Illumina sequencing information

Sample	CAV1015	CAV1016	CAV1374	CAV1411	CAV1492	CAV1596	CAV1741	P46212
Species	Koxy	Kpne	Koxy	Eclo	Smar	Kpne	Cfre	Ecol
Reference size (Mb)	6.58	5.60	7.23	5.01	5.83	5.62	5.39	5.25
Total number of contigs	6	4	12	3	6	5	7	2
Number of plasmids	5	3	11	2	5	4	6	1
Number of Tn*4401*-harbouring plasmid	1	1	1	1	1	2	1 (duplicate Tn*4401*)	0
Plasmid	12-114	44-90	2-333	34-91	3-200	3-97	2-130	144

sizes (kb)								
Illumina sequencing	Miseq-pe, 250bp,380bp insertion	Miseq-pe, 250bp,380bp insertion	Miseq-pe, 250bp,380bp insertion	Miseq-pe, 250bp,380bp insertion	Miseq-pe, 250bp,410bp insertion	Miseq-pe, 250bp,360bp insertion	Miseq-pe, 250bp,400bp insertion	Hiseq-pe, 150bp, 300bp insertion
Illumina coverage	117x	77x	47x	157x	73x	177x	176x	57x

Supplementary Methods

MinION long-read sequencing

Among the eight samples, six were sequenced in pairs using the 2D Native barcoding genomic DNA protocol (SQK-LSK208), and two were sequenced individually using the 2D Genomic DNA by ligation protocol (also SQK-LSK208). Below are library preparation details of each kit.

2D Native barcoding genomic DNA (SQK-LSK208) library preparation

All steps were performed according to Oxford Nanopore Technologies'(ONT, UK) protocols with minor modifications to accommodate the longer length of the fragments. Following fragmentation, end-repair and dA-tailing (New England Biolabs, USA) were performed in a combined reaction followed by clean-up with 0.4x AMPure XP beads (Beckman Coulter, UK). All incubation periods during clean-up steps were doubled. Unique barcodes were ligated onto each sample using Blunt/TA Ligase Master Mix (New England Biolabs, USA) and cleaned in 0.4x AMPure XP beads. Barcoded samples were pooled in equimolar amounts and barcoding adapters and HP tether ligated using Quick T4 DNA ligase (New England Biolabs, USA). Samples were cleaned using MyOne C1 streptavidin beads (Thermo Fisher Scientific, USA) to preferentially select DNA fragments with successful hairpin adapter ligation.

2D Genomic DNA by ligation (SQK-LSK208) library preparation

All steps were performed according to Oxford Nanopore Technologies'(ONT) protocols. A supplementary clean-up step following fragmentation was performed using 1.8x AMPure beads. End-repair and dA-tailing were performed in a combined reaction followed by clean-up with 0.4x AMPure XP beads. HP adapters and HP tether were ligated using Blunt/TA Ligase Master Mix and cleaned in MyOne C1 streptavidin beads to preferentially select DNA fragments with successful hairpin adapter ligation.

PacBio HGAP references

The reference assemblies of the eight isolates were obtained from the SMRT PacBio sequencing pipeline and post-assembly manual curations. The long sequencing reads were assembled using HGAP (Chin et al., 2013). Illumina short-read sequencing was used to recover small plasmids and to manually refine the HGAP assemblies (Sheppard et al., 2016, Stoesser et al., 2016)

Assembly methods

The command-lines/scripts for performing different assembly approaches are described below.

SPAdes

```
> $spadesPath -1 fastq/$SAMPLEID/reads1.fq.gz -2 fastq/$SAMPLEID/reads2.fq.gz -o $outDir    --careful -t $NTHREAD
```

HybridSPAdes

```
> $spadesPath -1 fastq/$SAMPLEID/reads1.fq.gz -2 fastq/$SAMPLEID/reads2.fq.gz -o $outDir --nanopore nanoporeOutput/$RUNID.fasta --careful -t $NTHREAD
```

npScarf

runNpscarf.sh

```
-------------
nanoporeOutputPrefix=$2
spadesOutputPrefix=$1
spadesAssembly=spadesOutput/$spadesOutputPrefix/contigs.fasta
nanoporeReads=nanoporeOutput/$nanoporeOutputPrefix.fastq
jsaBin=$PATH_TO_JSA_BIN
$jsaBin/jsa.seq.sort -r --input  $spadesAssembly --output
spadesOutput/$spadesOutputPrefix/contigs_sorted.fasta

spadesAssembly=spadesOutput/$spadesOutputPrefix/contigs_sorted.fasta
bwa index $spadesAssembly
bwa mem -t 2 -k11 -W20 -r10 -A1 -B1 -O1 -E1 -L0 -a -Y $spadesAssembly $nanoporeReads  |
$jsaBin/jsa.np.gapcloser --bamFile - --sequenceFile $spadesAssembly --prefix
npscarfOutput/$spadesOutputPrefix.$nanoporeOutputPrefix.npscarf.scaffolds
------------------------
```

plasmidSPAdes

```
$spadesPath -1 fastq/$SAMPLEID/reads1.fq.gz -2 fastq/$SAMPLEID/reads2.fq.gz -o $outDir
--careful -t $NTHREAD --plasmid
```

Canu

```
$canu -p $prefix -d canuOutput/$SAMPLEID    corMhapSensitivity=high -nanopore-raw
nanoporeOutput/$SAMPLEID.fasta genomeSize=5m -java=/usr/lib/jvm/java-8-openjdk-
amd64/jre/bin/java maxThreads=4
```

Pilon

```
sampleName=$1
sampleName1=$2
REF=canu_only/$sampleName.canu_only.fa
outBamPrefix=tmpDir/$sampleName.bwa
bwa index $REF
bwa mem $REF fastq/$sampleName1/reads1.fq.gz fastq/$sampleName1/reads2.fq.gz
|samtools view -Shu - |samtools sort -   $outBamPrefix
samtools index $outBamPrefix.bam
echo "run java and pilon"
java -Xmx16G -jar pilon-1.18.jar --output $sampleName.canu_pilon --outdir
canuOutput/$sampleName/ --genome $REF --bam $outBamPrefix.bam
```

Results

Figure S1. Read length distributions of different nanopore sequencing runs for 8 samples

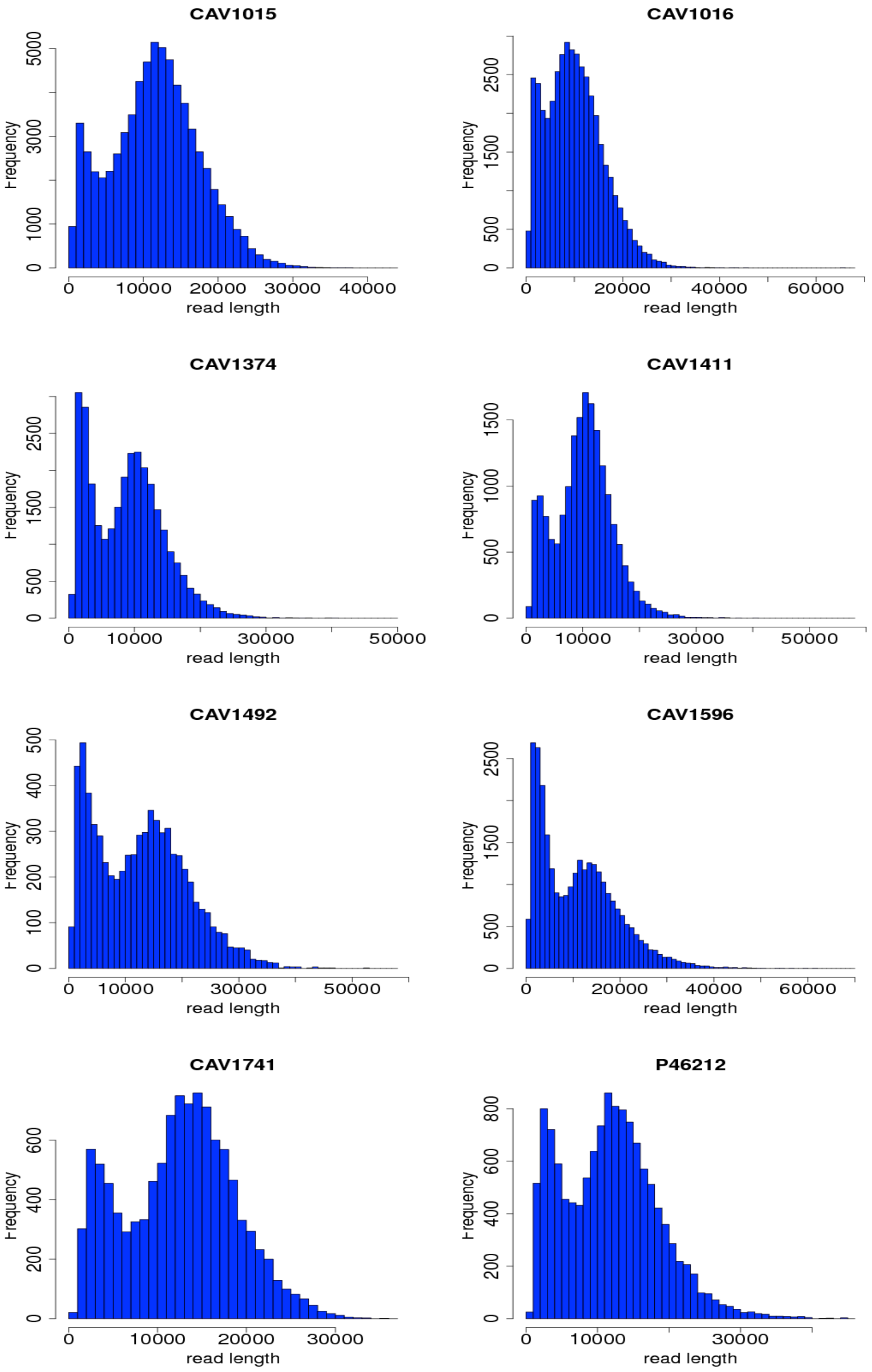

Figure S2. Alignments of different assemblers' longest contigs against chromosomal sequences of PacBio-based reference assemblies. In these chromosomal contig alignments, the black lines across the tracks show aligned regions between tracks. Repeat sequences (>5kb) are highlighted as multiple black lines derived from the same locations on a specific track (shorter repeats not shown).

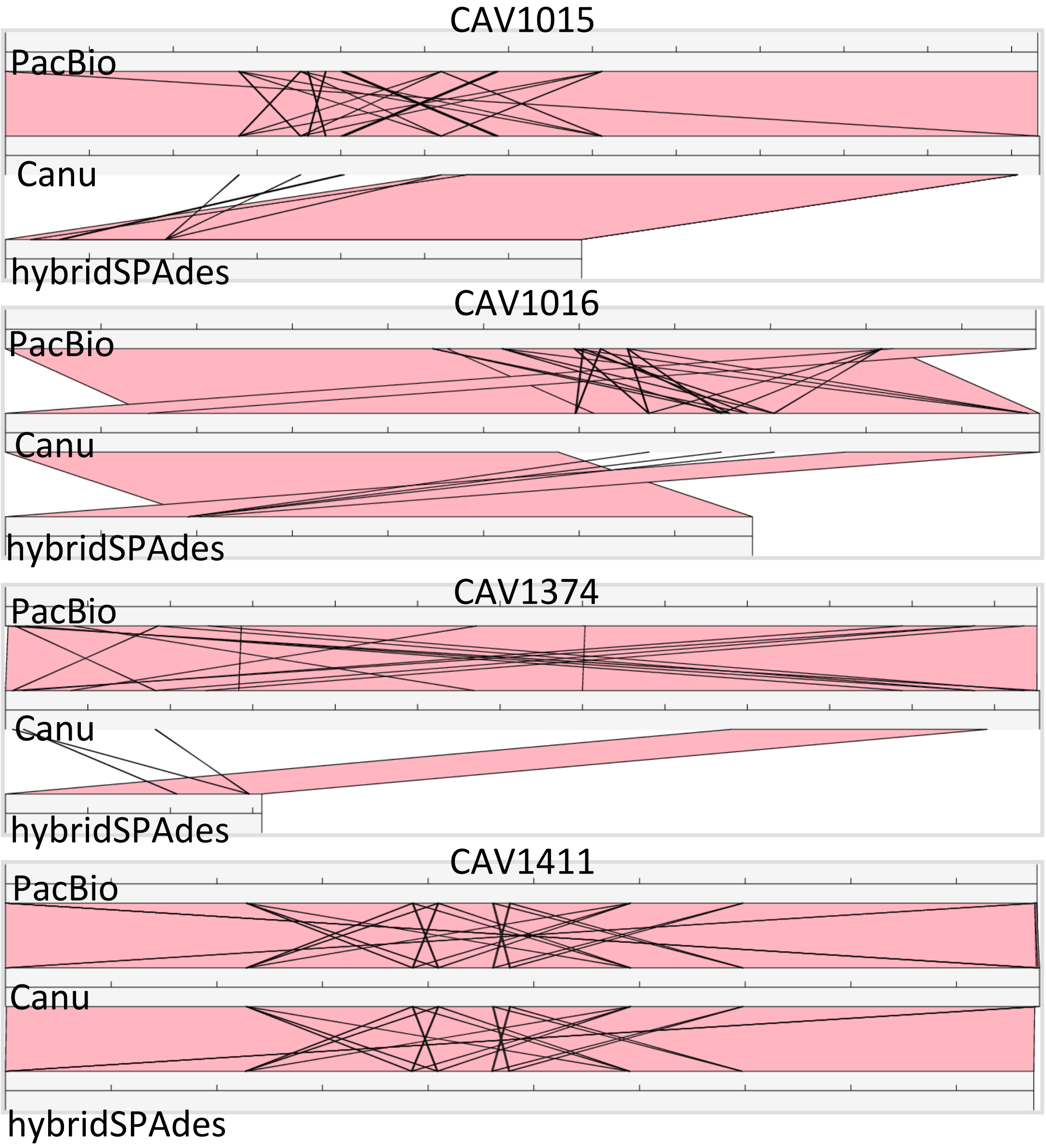

Supplementary Figure S2-a: Alignment of longest contigs from different assemblers against the PacBio-based chromosomal sequences, sample CAV1015, CAV1016, CAV1374, and CAV1411

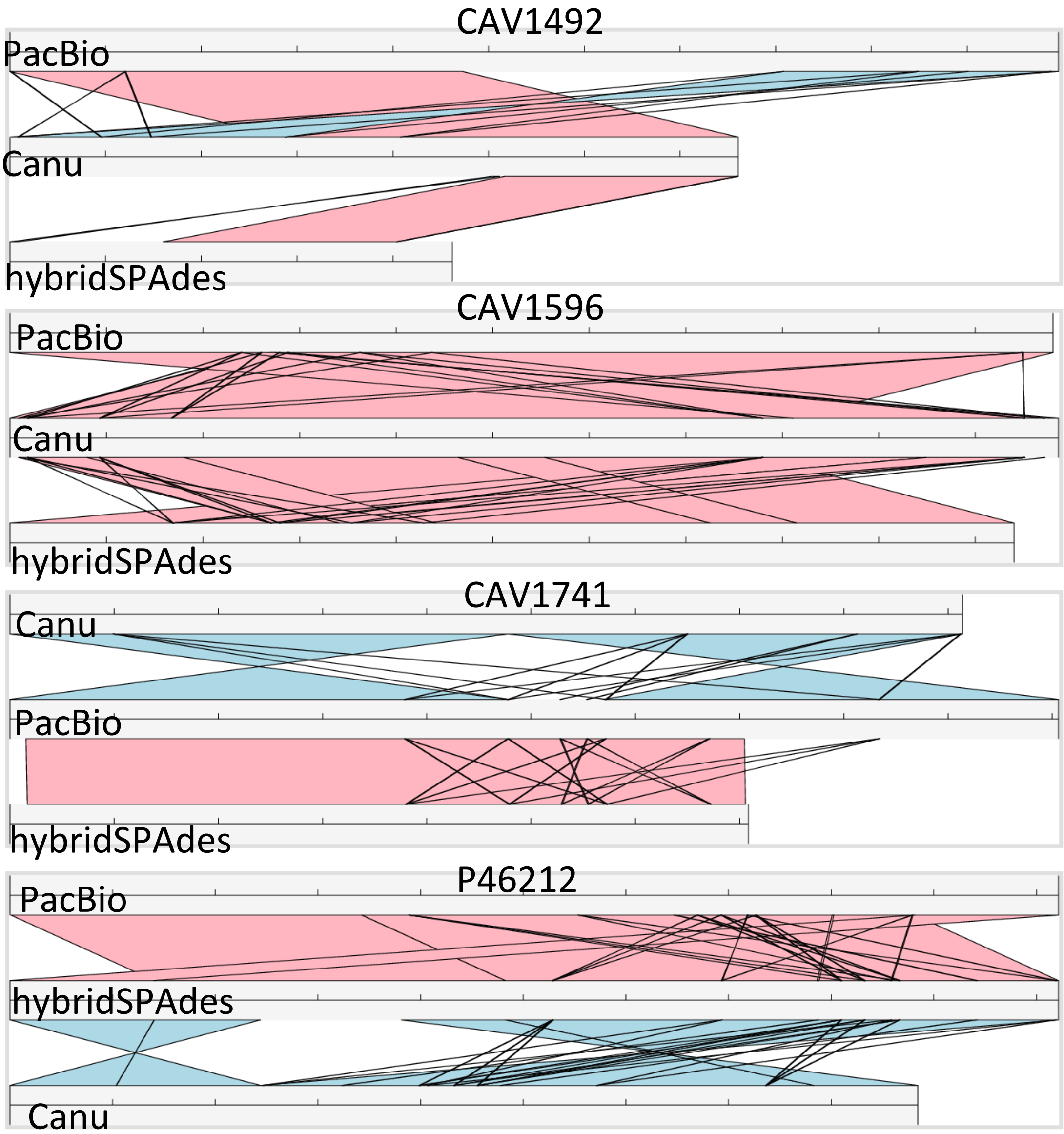

Supplementary Figure S2-b: Alignment of longest contigs from different assemblers against the PacBio chromosomal sequences, sample CAV1492, CAV1596, CAV1741, and P46212

Figure S3 Mummerplots of assemblies against the chromosomal sequences of PacBio-derived reference assemblies for isolates CAV1015, CAV1016, CAV1492, P46212

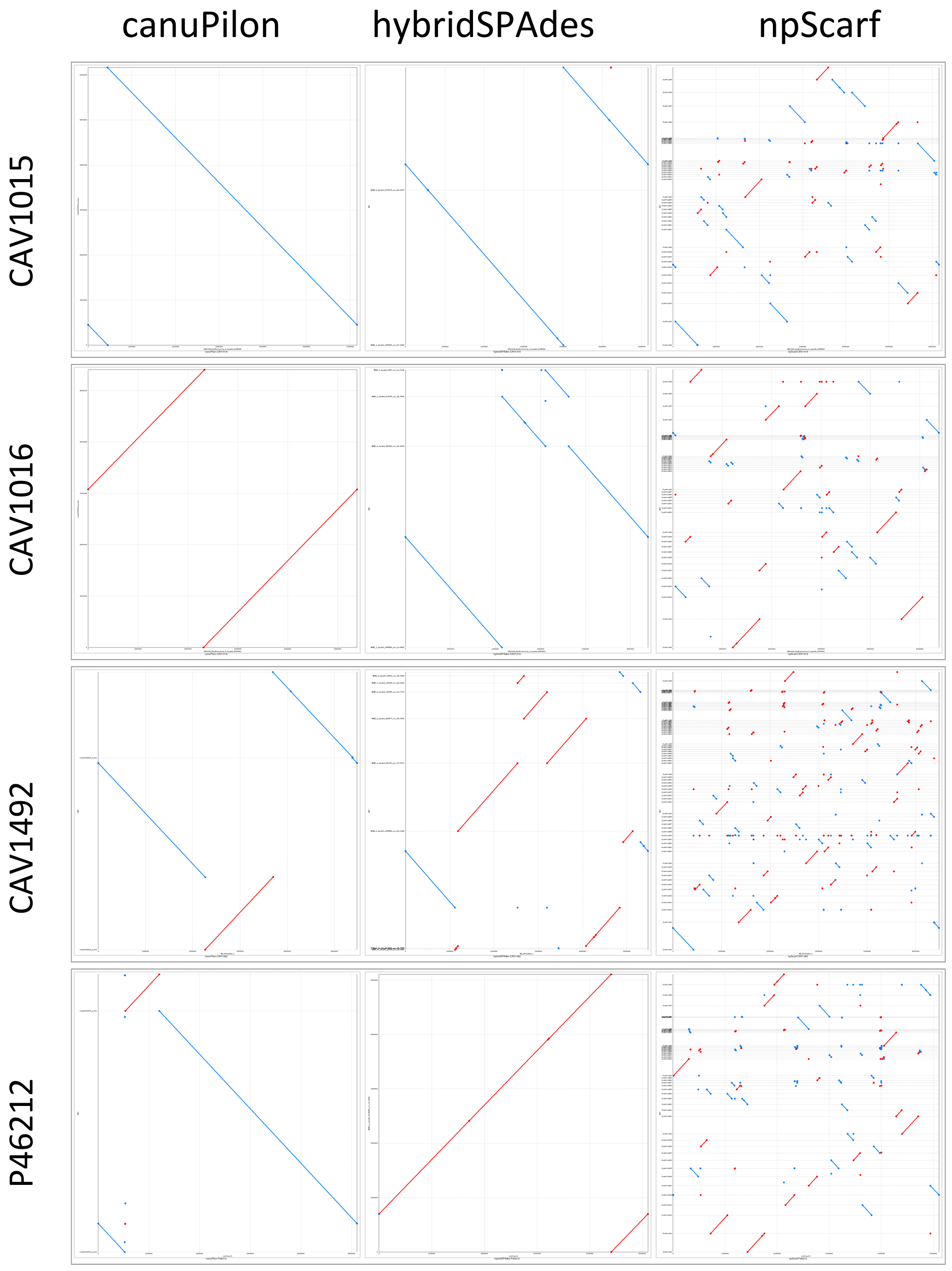

Figure S4. Mummerplots of assemblies against the chromosomal contigs of PacBio-based assemblies for isolates CAV1374, CAV1411, CAV1596 and CAV1741

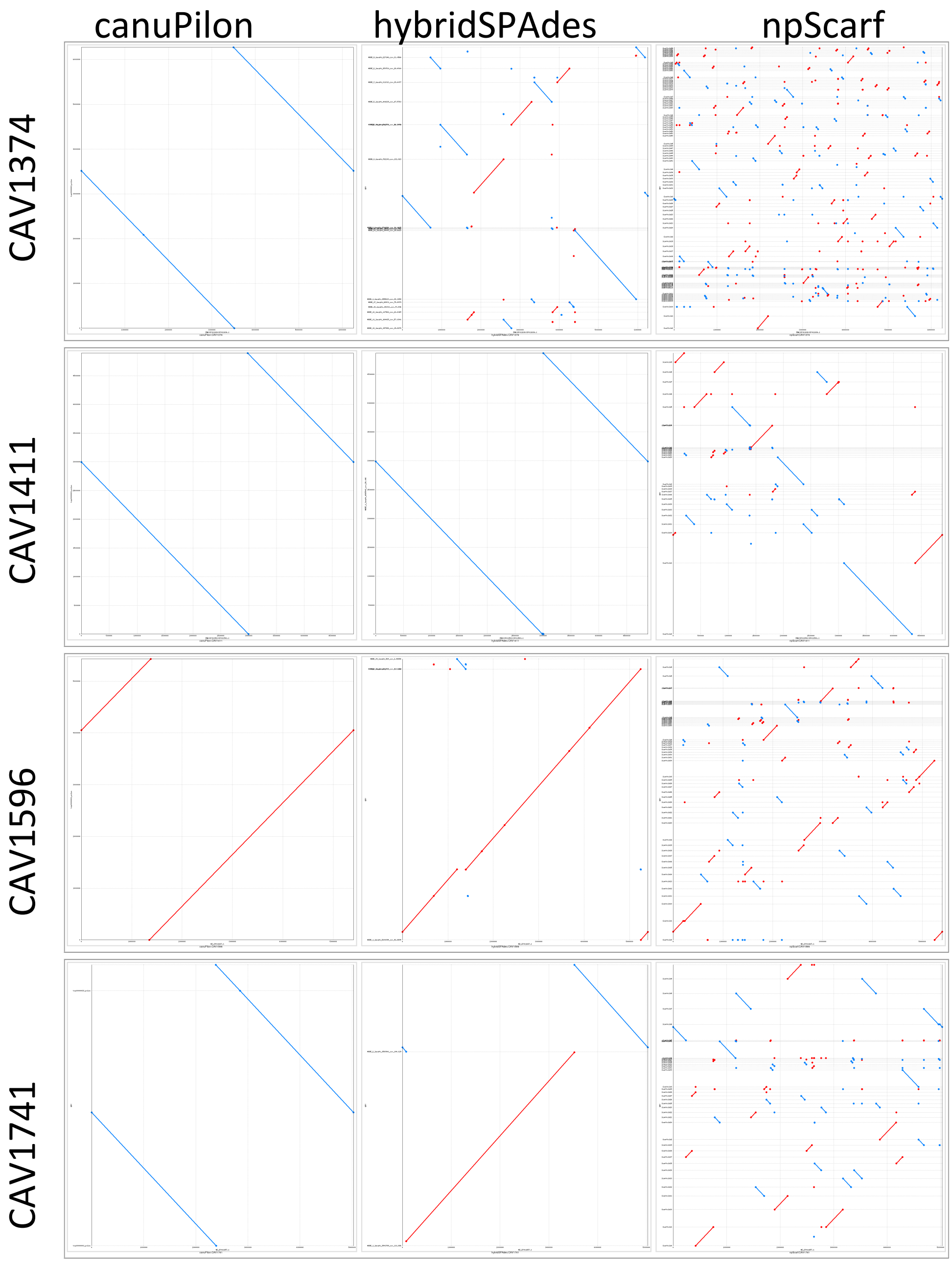

Figure S5. Mummerplots of different assemblers' contigs matching with plasmid sequences from PacBio-based assemblies for isolates CAV1015, CAV1016, CAV1492 and P46212

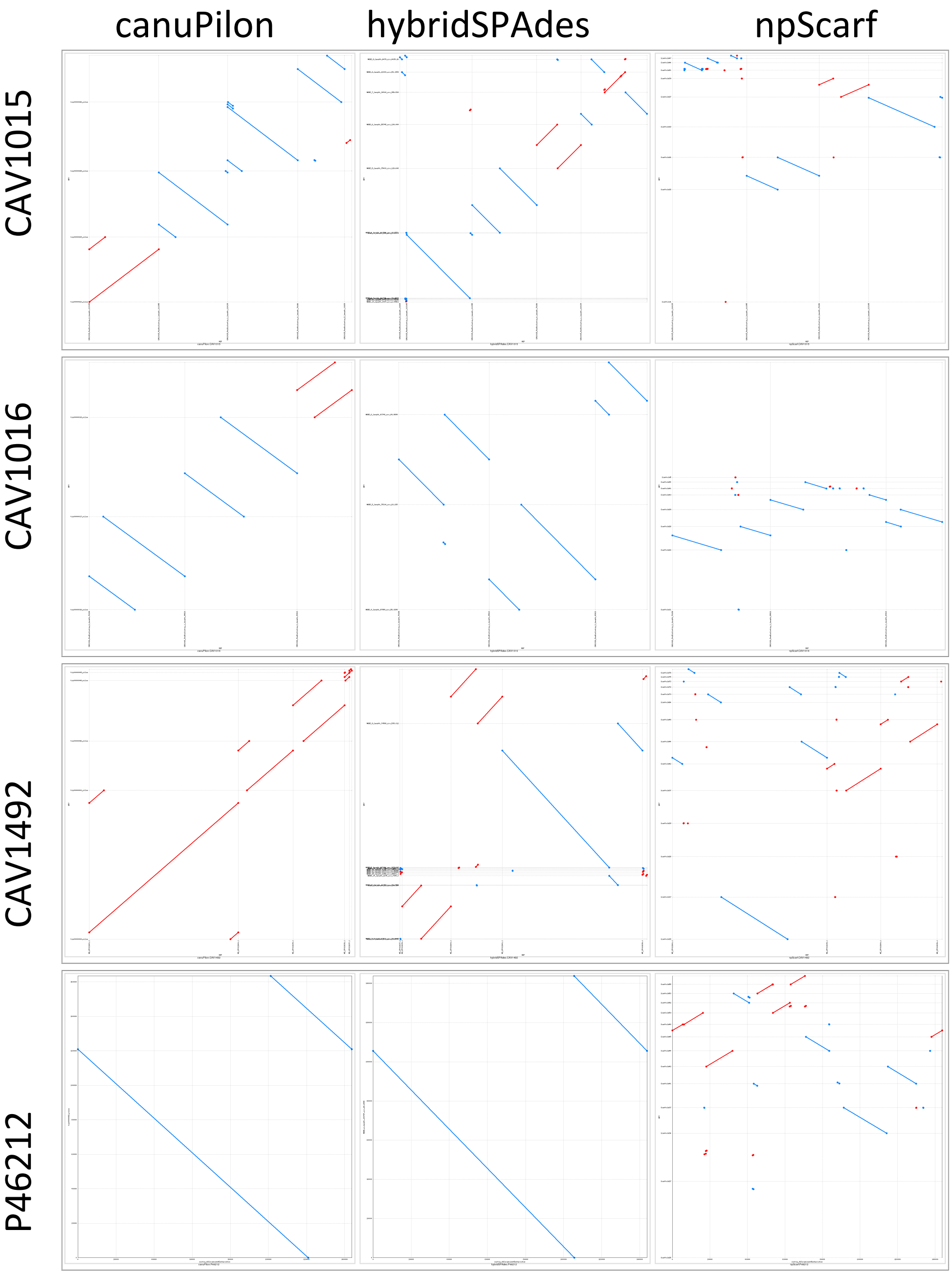

Figure S6. Mummerplots of different assemblers' contigs matching with plasmid sequences from PacBio-based assemblies for isolates CAV1411 and CAV1596

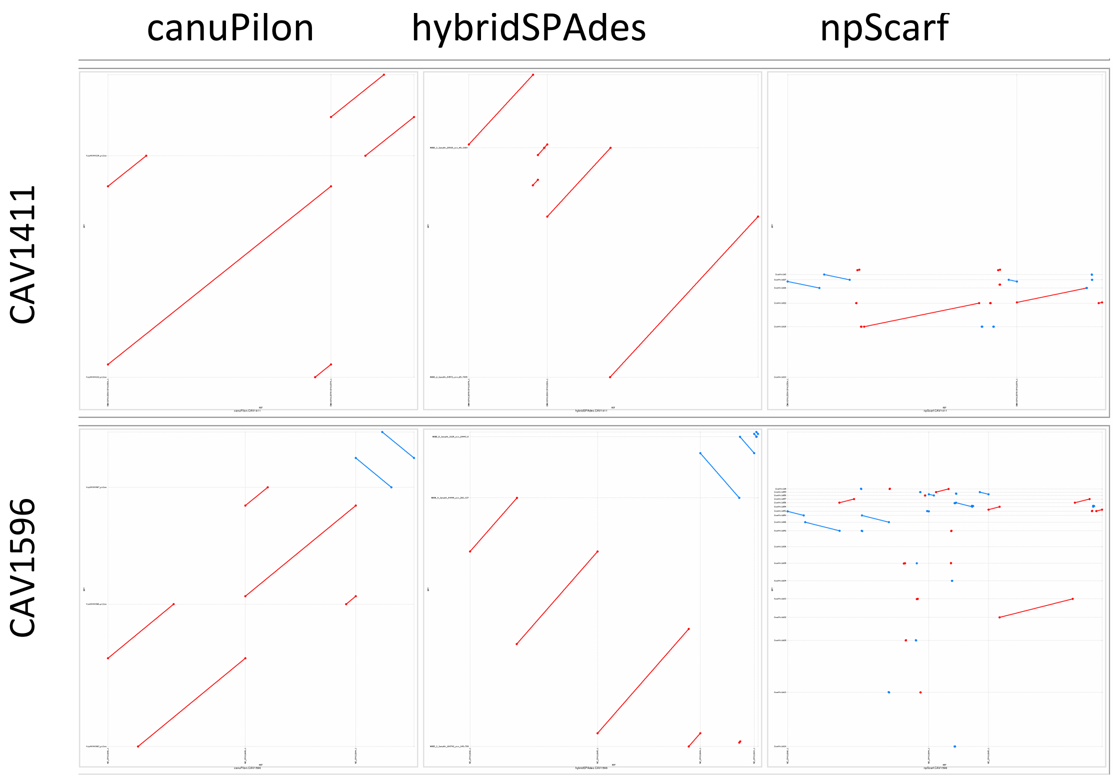

Figure S7. MinION read coverage of short plasmids not fully recovered or discovered by Canu assembler.

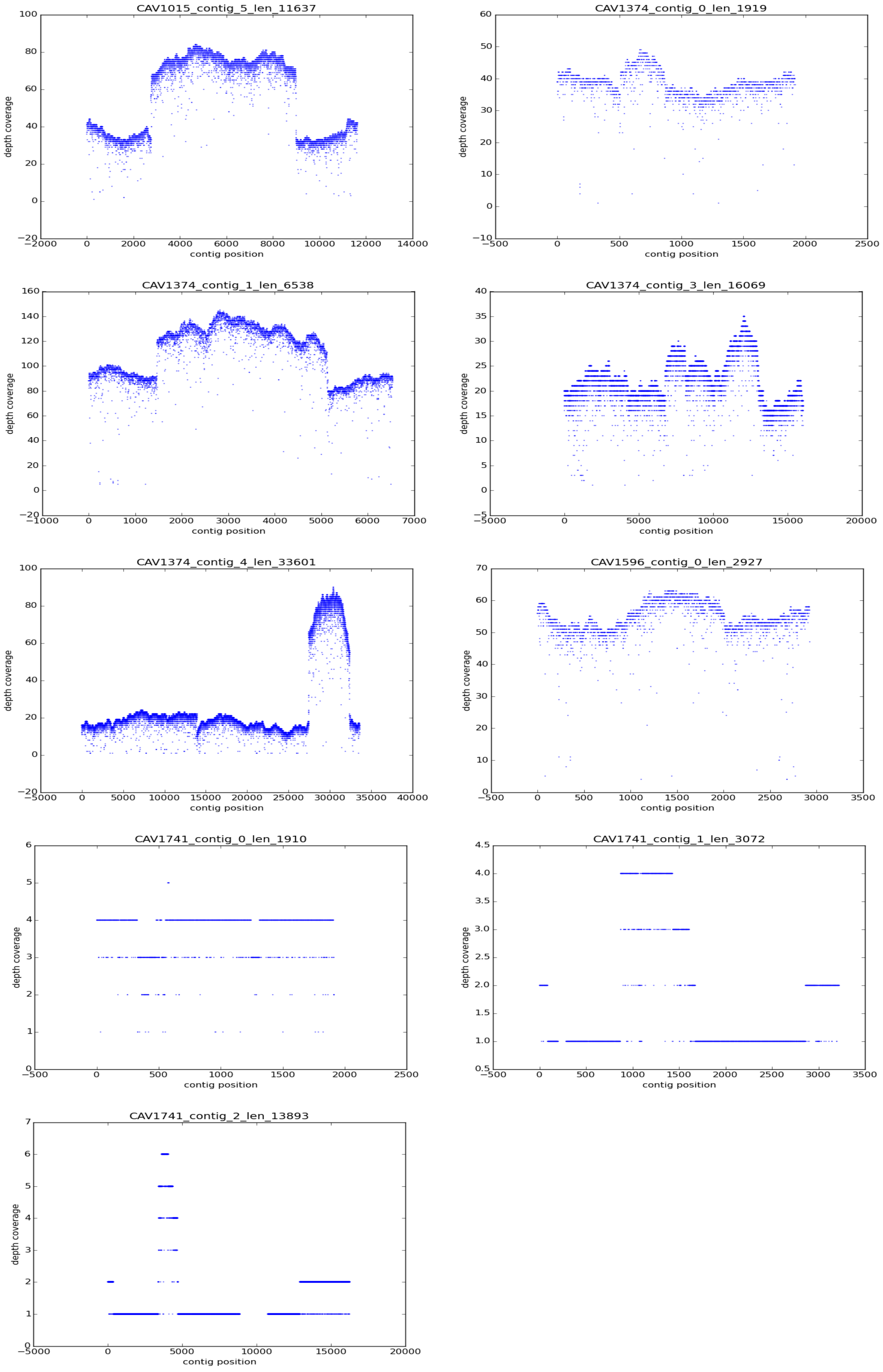

Figure S8. MinION read coverage of two long plasmids not resolved by Canu assembler

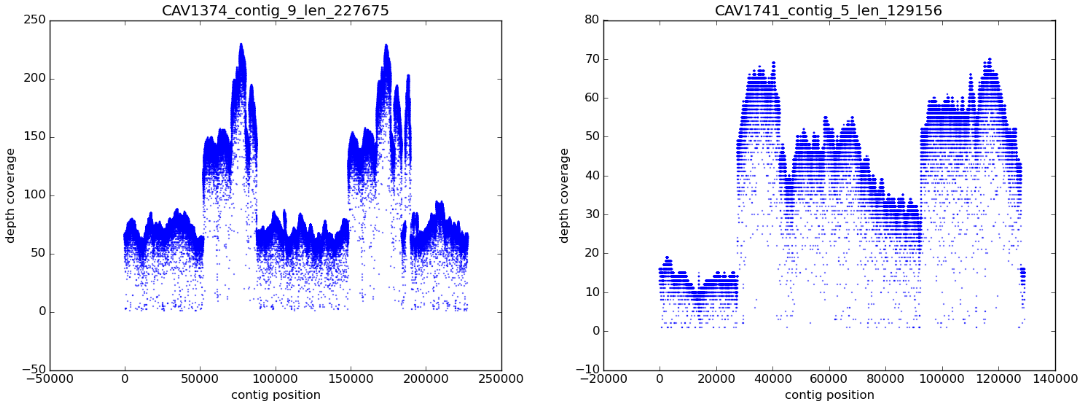

References

CHIN, C. S., ALEXANDER, D. H., MARKS, P., KLAMMER, A. A., DRAKE, J., HEINER, C., CLUM, A., COPELAND, A., HUDDLESTON, J., EICHLER, E. E., TURNER, S. W. & KORLACH, J. 2013. Nonhybrid, finished microbial genome assemblies from long-read SMRT sequencing data. *Nat Methods,* 10**,** 563-9.

SHEPPARD, A. E., STOESSER, N., WILSON, D. J., SEBRA, R., KASARSKIS, A., ANSON, L. W., GIESS, A., PANKHURST, L. J., VAUGHAN, A., GRIM, C. J., COX, H. L., YEH, A. J., MODERNISING MEDICAL MICROBIOLOGY INFORMATICS, G., SIFRI, C. D., WALKER, A. S., PETO, T. E., CROOK, D. W. & MATHERS, A. J. 2016. Nested Russian Doll-Like Genetic Mobility Drives Rapid Dissemination of the Carbapenem Resistance Gene blaKPC. *Antimicrob Agents Chemother,* 60**,** 3767-78.

STOESSER, N., SHEPPARD, A. E., PANKHURST, L., DE MAIO, N., MOORE, C. E., SEBRA, R., TURNER, P., ANSON, L. W., KASARSKIS, A., BATTY, E. M., KOS, V., WILSON, D. J., PHETSOUVANH, R., WYLLIE, D., SOKURENKO, E., MANGES, A. R., JOHNSON, T. J., PRICE, L. B., PETO, T. E., JOHNSON, J. R., DIDELOT, X., WALKER, A. S., CROOK, D. W. & MODERNIZING MEDICAL MICROBIOLOGY INFORMATICS, G. 2016. Evolutionary History of the Global Emergence of the Escherichia coli Epidemic Clone ST131. *MBio,* 7**,** e02162.
